# Introduction to the Special Issue on Ischemic Stroke in Children

**DOI:** 10.3390/children9060832

**Published:** 2022-06-03

**Authors:** Beata Sarecka-Hujar, Ilona Kopyta

**Affiliations:** 1 Department of Basic Biomedical Science, Faculty of Pharmaceutical Sciences in Sosnowiec, Medical University of Silesia in Katowice, 41-200 Sosnowiec, Poland; 2 Department of Pediatric Neurology, Faculty of Medical Sciences in Katowice, Medical University of Silesia in Katowice, 40-752 Katowice, Poland; ilonakopyta@autograf.pl

The occurrence of arterial ischemic stroke (AIS) is a serious medical problem due to the deleterious neurological consequences that affect the daily functioning of the patient as well as the costs of medical care and rehabilitation. AIS results from a multifactorial background in both children and in adults. However, the etiology of AIS in children differs significantly from that of adult AIS. In primary care, AIS is sometimes not taken into account when acute symptoms are observed in children. This also contributes to the delay in proper diagnosis and treatment. 

Particular attention, both in terms of the occurrence of the first incident of cerebral ischemia and possible recurrences in children, should be paid to the arteriopathy of cerebral vessels, especially focal cerebral arteriopathy of childhood (FCA). Genetic risk factors may be also involved in the etiology of childhood AIS, as indicated by the age of patients. Previously, several meta-analyses confirmed or denied the role of particular genetic polymorphisms in the development of childhood AIS [1,2,3]. The obtained results indicated that *MTHFR* 677C > T and *FII* 20210G > A polymorphisms may be considered as risk factors for the disease in pediatric patients [1,2]. In addition, non-genetic risk factors, including mainly biochemical ones, are of great interest, whereas environmental factors (which are common in adults) seem to have low or no clinical relevance in children. In many children suffering from AIS, no cause of the disease is being determined.

The clinical manifestation of ischemic stroke depends on the location of changes in the central nervous system. Circulatory disturbances in the intracranial artery and middle cerebral artery are characterized by paresis, less paralysis, central paralysis or facial nerve paralysis and aphasia. Focal symptoms are sometimes accompanied by headaches, less frequently epileptic seizures. In turn, in the clinical picture of disorders associated with the posterior vertebral region of the cerebral vascular system, features of the cerebellar syndrome will prevail. Thus, establishing the predictors (clinical, metabolic, or genetic) that increase the risk of stroke in children may be helpful in building strategies for secondary prevention. Additionally, knowledge on the predictors of poor post-stroke outcome in pediatric patients is of great relevance.

This Special Issue brings together two original research papers, as well as three literature reviews, on a variety of topics related to pediatric arterial ischemic stroke. The first original research by Kopyta et al. [4] carried out a retrospective analysis on clinical presentation of pediatric AIS and its consequences according to the neuroimaging results and location of ischemia in a group of 75 pediatric patients. The study demonstrates a correlation between the presence of a specific post-stroke result and the location of vascular or morphological changes observed in neuroimaging. In the vast majority of patients, magnetic resonance imaging (MRI) of the brain was performed, and nearly 59% of patients analyzed began with computer tomography (CT) as the first available urgent method. The authors showed that the localization of morphological changes correlated with intellectual retardation and epilepsy observed as a post-stroke outcome. Hemiparesis was found in 62% of children with changes in the temporal lobe. Post-stroke epilepsy and aphasia have been observed with the same frequency in patients with changes in the temporal lobe. In turn, 6% of patients with changes in the temporal lobe presented movement disorders other than hemiparesis, and 3% had intellectual retardation [4].

In the second original research, Antkowiak et al. [5] analyzed the clinical outcomes of 22 pediatric patients (mean age 11.9 years) who initially presented ruptured brain arteriovenous malformations (bAVMs). No procedural complications or re-bleeding were observed after interventional treatment. Overall, 19 patients (i.e., over 86%) showed good results on the modified Rankin scale (mRS 0–2) at discharge, while 3 patients (i.e., almost 14%) were classified as disabled (mRS 3). According to authors, the use of radiosurgery or embolization in the management of high-grade bAVMs may provide satisfactory results without a high risk of disability [5].

Among the three review papers published within the Special Issue, one investigated pediatric ischemic stroke in regard to initial approach and early management, as different clinical presentation in children may result in a delay in AIS diagnosis and in consequence may negatively influence the overall outcome [6]. The overall AIS-related mortality is progressively decreasing, which may result from generally better access to imaging methods, especially MRI, implementations of protocols on pediatric AIS, the establishment of modern centers for pediatric AIS, and guidelines for pediatric AIS diagnosis and management. Next, review by Kopyta et al. [7] discussed the current literature data on the incidence of early death in children suffering from AIS. Authors analyzed also risk factors for early death in pediatric patients with stroke. Analysis of available data showed that the incidence of in-hospital deaths in children with AIS ranged from 2.6 to 14% and the possible risk factors for mortality after AIS in pediatric patients may be identified from the following: patient’s ethnicity, etiopathogenesis and underlying diseases, or age at stroke onset. The last review paper by Sarecka-Hujar et al. [8] concerns the important topic on the role of sex in AIS occurring in young patients (i.e., children and young adults). Previous data suggested that male pediatric patients are more prone to AIS, and male gender predominance is especially observed in newborns. To understand this phenomenon in children, the potential relationship with trauma and arterial dissection is raised while in young adults, men suffer from AIS more frequently than women, probably due to the protective role of estrogen. The authors pointed out the disparities in the frequencies of particular symptoms of AIS in both children (e.g., central type facial nerve palsy in boys while in girls no specific symptoms were identified) and young adults (e.g., dysarthria and swallowing deficits in men while migraine in women). Additionally, poststroke consequences may also differ between sexes in both children (e.g., stroke recurrence and cognitive deficits in boys while seizures in girls with certain stroke type) and young adults (e.g., cumulative 20-year mortality in men while poorer outcome defined as 3–6 in modified Rankin Scale in women). In addition, some differences between sexes were reported in young patients in regard to genetic risk factors for AIS [8].

In conclusion, this Special Issue emphasizes that there is a need for further research on ischemic stroke in children. All these papers should contribute to increasing our knowledge about the causes, manifestations, and management of AIS in pediatric patients.

## References

[1] Sarecka-Hujar B., Kopyta I., Pienczk-Reclawowicz K., Reclawowicz D., Emich-Widera E., Pilarska E. (2012). The TT genotype of methylenetetrahydrofolate reductase 677C>T polymorphism increases the susceptibility to pediatric ischemic stroke: Meta-analysis of the 822 cases and 1552 controls. Mol. Biol. Rep..

[2] Sarecka-Hujar B., Kopyta I., Skrzypek M., Sordyl J. (2017). Association between the 20210G>A Prothrombin Gene Polymorphism and Arterial Ischemic Stroke in Children and Young Adults-Two Meta-analyses of 3586 Cases and 6440 Control Subjects in Total. Pediatric Neurol..

[3] Sarecka-Hujar B., Kopyta I., Skrzypek M. (2019). Lack of Associations between PAI-1 and FXIII Polymorphisms and Arterial Ischemic Stroke in Children: A Systematic Review and Meta-Analysis. Clin. Appl. Thromb. Hemost..

[4] Kopyta I., Sarecka-Hujar B., Raczkiewicz D., Gruszczyńska K., Machnikowska-Sokołowska M. (2021). Assessment of Post-Stroke Consequences in Pediatric Ischemic Stroke in the Context of Neuroimaging Results-Experience from a Single Medical Center. Children.

[5] Antkowiak L., Putz M., Rogalska M., Mandera M. (2021). Multimodal Treatment of Pediatric Ruptured Brain Arteriovenous Malformations: A Single-Center Study. Children.

[6] Klučka J., Klabusayová E., Musilová T., Kramplová T., Skříšovská T., Kratochvíl M., Kosinová M., Horák O., Ošlejšková H., Jabandžiev P. (2021). Pediatric Patient with Ischemic Stroke: Initial Approach and Early Management. Children.

[7] Kopyta I., Cebula A., Sarecka-Hujar B. (2021). Early Deaths after Arterial Ischemic Stroke in Pediatric Patients: Incidence and Risk Factors. Children.

[8] Sarecka-Hujar B., Kopyta I. (2021). The Impact of Sex on Arterial Ischemic Stroke in Young Patients: From Stroke Occurrence to Poststroke Consequences. Children.

